# Unlocking the Therapeutic Potential of Freshwater Sapropel Extracts: In Vitro Analysis and Antioxidant Profiling for Skincare Applications

**DOI:** 10.3390/medicina60040546

**Published:** 2024-03-27

**Authors:** Aneka Kļaviņa, Jeļena Reste, Inese Mārtiņsone, Ivars Vanadziņš, Anna Lece, Ilona Pavlovska

**Affiliations:** 1 Department of Occupational and Environmental Medicine, Riga Stradiņš University, LV-1007 Riga, Latvia; aneka.klavina@rsu.lv (A.K.);; 2 Institute of Occupational Safety and Environmental Health, Riga Stradiņš University, LV-1007 Riga, Latvia; 3 Scientific Laboratory of Biochemistry, Riga Stradiņš University, LV-1007 Riga, Latvia

**Keywords:** antioxidants, biological polyacids, cell growth, humic acid, freshwater sapropel, fulvic acid, in vitro

## Abstract

*Background and Objective:* Sapropel, a biologically active sedimentary deposit, is high in organic matter and minerals and has been shown to offer health benefits. Its constituents, humic acid (HA) and fulvic acid (FA), have been found to have some therapeutic applications. The aim of this study was to determine the potential therapeutically significant properties of freshwater sapropel extracts: their polyacid content, antioxidative (AO) status, and biological activity in cell culture. *Materials and Methods:* Freshwater lakes from the southeast region of Latvia were investigated layer by layer. The total organic carbon (TOC) was determined through combustion using the catalytic oxidation method, HA and FA were measured via acid perspiration, and the total polyphenol content (TPC) and total antioxidant status (TAS) was analysed spectrophotometrically. Sapropel extracts’ regenerative abilities were tested in vitro using a Cell-IQ real-time monitoring system on mouse BALB/c 3T3 fibroblasts and human keratinocyte HaCaT cell lines. Cytotoxicity was measured through neutral red uptake assessment as a concentration-dependent reduction in the uptake of neutral red dye relative to a vehicle control or untreated cells. *Results:* The highest AO activity was observed in sapropel extracts with elevated concentrations of HA and TPC from Audzelu Lake (1.08 ± 0.03 mmol/L), and the lowest activity was found in extracts from Ivusku Lake (0.31 ± 0.01 mmol/L). Correspondingly, the concentrations of HA in Audzelu and Ivusku Lakes were recorded as 45.2 and 27.4 mg/g, respectively. High concentrations of HA promoted in vitro cell growth upon short-term exposure (up to 6 h). *Conclusions:* The results show that high TPC correlates with AO status and sapropel extracts with higher concentrations of HA exhibit greater AO activity and promote in vitro cell growth, suggesting a perspective use for short-term topical therapeutic skin applications. However, higher concentrations over longer durations showed cytotoxic effects, indicating the need for further investigation.

## 1. Introduction

The increased interest in sapropel use for healthcare, cosmetics, and the pharmaceutical industry means that better knowledge is required regarding sapropel properties and the variations between various sources (the origin lake and depth from which sapropel is extracted).

Sapropel mud is a multi-component system comprising organic mineral complexes and organic matter and is used in healing procedures [1]. Sapropel is a semi-renewable resource from subterranean depths, as it consists of fine-grained and friable continental water sediments with an organic matter content of no less than 15%, mainly consisting of aquatic animal and plant residues. Sapropel is found in most lakes in Latvia as sediment, and many types of mire also contain sapropel under the peat layer [2,3]. There are several types of sapropel in Latvia—organic sapropel, carbonated sapropel, silica sapropel, and mixed-type sapropel [2]. Sapropel types differ in their concentration of biologically active components and oxide concentration. The sapropel pH level is around 6–8, and the environmental pH reaction is neutral; if pH levels are higher, then sapropel has a high mineral content. The sapropel type can be established based on the ash content [2,3].

Sapropel has been used in resorts with mineral springs, in facilities devoted to relaxation and health (spas), and in aesthetic medicine; its usage has grown due to an increased interest in natural remedies [4]. During sapropel application in balneotherapy, some chemicals penetrate the skin [5]. Sapropel sediments have an antioxidant effect that smooths wrinkles, prevents new wrinkles, and smooths the skin structure, as well as removing swelling and strengthening nails and hair [4,5]. Therefore, the therapeutic effect of sapropel helps to maintain the cellular structure of the skin [6,7] and restore immunity and helps with acne, rashes, and dermatitis [8,9]. In addition, balneotherapy has shown healing capabilities in the treatment of various musculoskeletal and rheumatologic diseases [10].

Sapropel sediments consist mainly of humic substances, which are organic macromolecules [11], major parts of which are humic acid (HA) and fulvic acid (FA) [12,13]. Organic acids are the most important biologically active substances in sapropel sediments. HA and FA participate in healing processes. Organic acids consist of both aliphatic and aromatic structures with different functional groups [8]. The molecular structure of HA is an aromatic polymer containing carboxyl, hydroxyl, methyl, and phenolic groups [14,15]. The average molecular weight is 6500 Da. The molecular weight of FA is from 400 to 2000 Da, depending on the source [16]. The main structural skeleton comprises poly-functional groups with polypeptides and polysaccharides bound to them [14,17]. The HA molecule reacts with the receptors in the dermis due to the aromatic nucleus and functional groups. The anti-inflammatory effect of HA has been supported by its inhibition of the production of inflammatory cytokines. HA produces a therapeutic effect by inhibiting the lipoxygenase pathways of the arachidonic acid cascade [8,14,18]. Arachidonic acid is a component of the cell membrane and a substrate for the synthesis of eicosanoid-based inflammation mediators such as leukotrienes, thromboxane, and prostacyclin. HA, as well as FA, has been found to suppress the heat-induced arachidonic acid release of human promonocytic cells. It is known that wound-healing processes require additional oxygen, and this demand appears in the first minutes after phagocytosis begins. HA has the ability to generate extra oxygen. Based on the molecular size, HA cannot penetrate the deeper layers of the skin, but it can act as a wound-healing agent, while FA, which has a smaller molecular size, can penetrate deeper into the skin and help cell growth by acting on healthy cells. FA’s major therapeutic effect relates to the activation of skin metabolism and regeneration processes [6,19,20].

It is important to obtain sapropel extracts to more easily achieve biologically active substance penetration through skin tissues and for therapeutic effects. HA and FA may be extracted for further use from sapropel sediments, and it is necessary to determine their content and full antioxidant properties [21].

This study was established as a part of the European Social Fund project, ‘Analysis of characteristics of medical sapropel and its usage for medical purposes and elaboration of industrial extraction methods’. Previously, the average depth of the lake water layer, sapropel layer depth, lake surface area, lake bottom structure, and surrounding and populated areas, as well as the hydrological regime and the sedimentological description of the mud section, have been described [3]. The determination of metals such as lead, cadmium, nickel, cobalt, copper, antimony, and chromium in sediments were measured in all samples, but none of the metals exceeded the maximum acceptable levels given by the Scientific Committee on Consumer Safety (SCCS) [3,22,23].

The use of sapropel in medicine requires sapropel samples to be free from pesticide residues and their contents to follow regulatory requirements. The best-known pesticide in the world is dichlorodiphenyltrichloroethane (DDT). Some water and sapropel samples at different depth levels showed a minor presence of the DDT pesticide and its decomposition product dichlorodiphenyldichloroethylene (DDE). The concentrations of DDE/DDT found in surface water were, in general, lower than those found in sediments. The highest levels of DDE/DDT were found at all depths in Mazais Kivdalova and Zeilu and in some extraction sites at Audzelu Lake, but the amount of DDE/DDT was below the limit of quantification. The current study continued the research on sapropel properties. Determining the HA and FA content, antioxidants, and biological activity of the sapropel extracts from separate lakes was the main aim [3]. Five lakes located in the eastern part of Latvia in the Latgale Upland were studied.

## 2. Materials and Methods

### 2.1. Exploration of Sapropel Samples

Eastern Latvian lakes with the following geographic coordinates were chosen for sapropel extraction: Audzelu Lake (56°15′35.4″ N 27°58′36.2″ E), Dunaklu Lake (56°33′23.1″ N 27°42′49.6″ E), Ivusku Lake (56°24′13.6″ N 27°20′51.4″ E), Zeilu Lake (56°30′40.3″ N 27°40′03.5″ E), and Mazais Kivdalovas Lake (56°32′58.0″ N 27°42′44.6″ E). These lakes have a glacial origin and were formed by a glacier and its melting waters. All the lakes have similar formation conditions (originating in Latgale Upland); however, they are in separate areas of the upland. Mazais Kivdalovas, Zeilu, and Dunaklu Lakes are located in the Rezeknes Lowlands, Ivusku Lake is located in the Raznava Hills, and Audzelu Lake belongs to the Dagda Hills. The freshwater sapropels in these lakes started to form during the Holocene.

**Figure 1:**
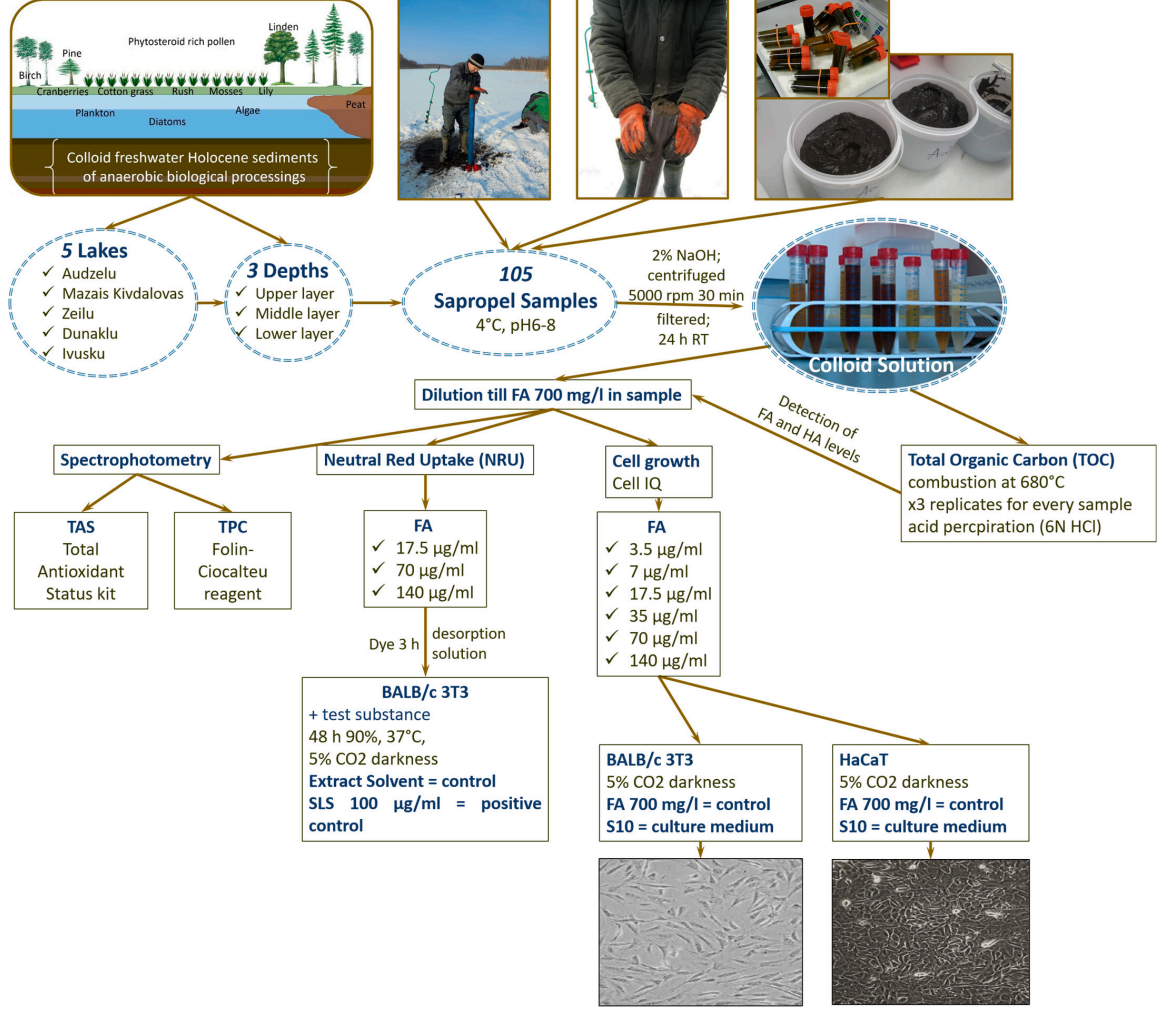
Scheme of the study design (FA, fulvic acid; TAS, total antioxidant status; TPC, total polyphenol content; BALB/c 3T3, cell culture of mouse dermal fibroblasts; HaCaT, aneuploid immortal keratinocyte cell line from adult human skin; SLS, sodium laureth sulphate).

During sample exploration, the sapropel sediment layer thickness, type of sapropel, and agricultural history of the territory next to the lake, as well as potential industrial waste exposure, were assessed [3]. Three depths of sediment (upper, middle, and lower layers, based on extraction depth from the top of the mud layer) were considered for exploration. Sediment samples were collected separately with semi-cylindrical chambers and kept in closed neutral plastic containers without oxygen access to prevent oxidation of samples. Sapropel sediments were stored at 4 °C in the dark until later analysis. In this study, 105 freshwater sapropel samples were obtained (each sample was 3 to 5 L). The scheme of the study design is shown in Figure 1.

### 2.2. Extraction of HA and FA from Sapropel

Sapropel extract was obtained using a solid–liquid extraction process with an alkaline solution [21]. Sapropel sediments, with 2% NaOH (AC, Sigma-Aldrich, Steinheim, Germany) solution, were stirred for 24 h in a closed reactor at room temperature; the mixture was centrifuged at 5000 rpm for 30 min and then filtered. After the addition of the sodium hydroxide solution, the pH rose from neutral to pH 10, and the chemical processes of cell disintegration began; stirring helped to mix the basic alkaline solution with the sediments, and a colloidal solution was formed. After centrifugation, sand particles and insoluble matter precipitates were discarded. Sapropel extract was stored at 4 °C for analysis. Prior to analysis, the HA and FA concentrations were determined for each sample through total organic carbon detection and acid perspiration. For further test purposes, each sample was diluted or concentrated to reach an FA concentration of 700 µg/mL to allow for a better comparison between lakes.

Determination of pH for each extract was performed using digital pH meter (WinLab Data Line pH/mV meter, Clausthal-Zellerfeld, Germany). The electrodes were inserted into 10 mL of sapropel extract for 10 min prior to taking the readings at room temperature, measurements were conducted in triplicate, and average value calculated to detect any pH fluctuation with time.

### 2.3. Determination of Total Organic Carbon (TOC), FA, and HA Concentration

The TOC concentration represents the fraction of organic matter that has escaped mineralisation during sedimentation. The TOC concentration often varies; it indicates changes in an organic deposition under different sedimentary conditions.

The TOC was determined through the combustion of the samples at 680 °C using the catalytic oxidation method (Shimadzu, TOC-V model). All analyses were repeated several times (n = 3) to check the repeatability and validity of the results. Each sapropel sample was measured in three replicates, and an average of three results was obtained.

Acid perspiration was chosen to determine the concentration of HA and FA. Extraction was performed for a dried and crushed sapropel sample (2 g) with 50 mL of 2% NaOH solution, which was stirred for 24 h to achieve perspiration and then centrifuged (30 min) for separation. The test solution containing HA and FA was acidified with 6 N HCl (AR, Sigma-Aldrich, Steinheim, Germany); after 16 h, high-speed centrifugation was used for the final separation. The mixture was filtered through a 0.45 µm membrane filter. The filtrate was analysed for TOC (TOC_filtrate_). Since the mixture was only composed of HA and FA, after acidification, the HA was precipitated and the solution phase contained only FA (C_FA_ = TOC_fitrate_) based on the definition of the HA and FA. It follows that the difference between the TOC content (TOC_total_) and TOC_filtrate_ will be equal to the HA concentration (C_HA_ = TOC_total_ − TOC_fitrate_).

### 2.4. Antioxidant (AO) Activity

The AO activity of sapropel extract with an FA concentration of 700 µg/mL was assessed. AO activity was measured in vitro using spectrophotometric methods to determine total antioxidant status (TAS) and total polyphenol content (TPC) due to polyphenols usually contributing greatly to antioxidant properties of extracts.

#### 2.4.1. Measurement of the TAS

The TAS in the sapropel extract was measured using the Total Antioxidant Status commercial assay kits (Cat. NX2332, Randox Laboratories Ltd., Crumlin, UK) adapted to the RX Daytona™ automated chemistry analyser (Randox Laboratories Ltd., Crumlin, UK) following the manufacturers’ instructions. In brief, the assay was based on the formation of ferryl myoglobin radical from metmyoglobin and hydrogen peroxide, which then oxidised ABTS (2,2′-azino-bis(3-ethylbenzothiazoline-6-sulfonic acid)) to produce the radical cation (ABTS•+), a green soluble chromogen, determined spectrophotometrically. Antioxidant scavenging led to the formation of cation radicals in a concentration-dependent manner, with a proportional decrease in colour intensity. Assay results are expressed as mmol/L. Total antioxidant measurement considers the cumulative effect of all antioxidants present in the extract under investigation.

#### 2.4.2. Total Polyphenol Content (TPC)

The TPC in the sapropel extract was determined spectrophotometrically via UV–Visible spectrophotometer UV-Vis Varian Cary 50 (Varian Australia Pty Ltd., Mulgrave, Australia), applying the widely used Folin–Ciocalteu method [24,25]. The basis of the method is the oxidation of the phenol –OH groups in the reaction with the Folin–Ciocalteu reagent, which is a mixture of phosphomolybdate and phosphotungstate used for the colorimetric in vitro assay of phenolic and polyphenolic antioxidants. It produces a blue colouration with an absorption at 765 nm. The colour of the solution is proportional to phenol concentration. The reducing capacity of the Folin–Ciocalteu reagent depends on the presence of –OH groups in polyphenols. First, 2.5 mL of 10% Folin–Ciocalteu reagent was added to 0.5 mL sapropel extract sample and mixed and incubated at room temperature for 3–8 min, then 2.0 mL of 7.5% sodium bicarbonate was added and mixed and incubated for 30 min at room temperature. The reading was taken at 765 nm against the ‘blank’ sample. The content of phenolic compounds in the extract was expressed as gallic acid (AR, Sigma-Aldrich, St. Louis, MO, USA) equivalents (μg GAL/mL). The gallic acid was used to set up a standard curve (concentration 100, 50, 25, 12.5, 6.25 µg GAL/mL). Samples were analysed in triplicate.

### 2.5. Neutral Red Uptake (NRU)

NRU was measured, and the relative comparison of the sapropel extract effect on NRU was assessed. The NRU test was carried out according to the Organisation for Economic Co-operation and Development (OECD) recommendations for NRU protocol [26].

Neutral red (NR) dye captured by a viable cell was released during the desorption step, which resulted in the well staining red, and this colour change was spectrophotometrically quantified in the plate reader (Tecan Infinite F50 with Magellan Tracker software, Tecan, Switzerland, https://lifesciences.tecan.com/products/microplate_readers/infinite_f50 [accessed 26 January 2024]) at 540 nm. Cytotoxicity was expressed as a concentration-dependent reduction in the uptake of NR dye relative to vehicle control or untreated cells, respectively; the more intense the colour of the well, the more viable cells there were.

Routinely, BALB/c 3T3 fibroblasts (Bagg Albino mouse line developed by S.A. Aaronson and G.T. Todaro in 1968) were cultivated in a monolayer to ~80% confluence in cell culture flasks (SARSTEDT, TC Flask T75, Stand., Vent. CAP.) in an incubator with 90% humidity at 37 °C with 5% CO_2_ in darkness, with cells visually inspected every day. The growing medium (S10) consisted of 90% of Dulbecco’s Modification of Eagle’s Medium (DMEM) (Millipore VLE Dulbecco’s MEM) accompanied by a 1% mixture of penicillin and streptomycin (Sigma-Aldrich, Penicillin–Streptomycin, Steinheim, Germany) and 10% fetal bovine serum (FBS) (SIGMA, Fetal Bovine Serum, Steinheim, Germany). Subcultivation or recultivation of cells was carried out if the confluence was 50–80%; 1x phosphate-buffered saline (PBS, Sigma-Aldrich, Steinheim, Germany) was used for cell washing, and 0.25% trypsin–EDTA solution (SIGMA, Trypsin–EDTA, Steinheim, Germany) was used to detach cells from the surface. NRU was assessed by using a slightly modified version of the test protocol. It was observed that sometimes, in some samples, a precipitate formed after defrosting; in these cases, they were centrifuged and filtered once again through 0.2 μm filters.

For the NRU test, 100 μL of S10 medium with 3000 cells per well was cultured in the 60 middle wells on the 96-well plate (Microtest Plate 96 Well), and 100 μL of S10 medium without cells were added to the wells along the plate perimeter. The plate was incubated for 24 h in an incubator with 90% humidity at 37 °C and 5% CO_2_ in darkness. After 24 h, the medium was gently removed via careful inversion of the plate over the appropriate container (i.e., ‘dumped’) and blotted on sterile paper towels. Fresh medium containing test substances or controls was added to all wells and incubated for 48 h in an incubator with 90% humidity at 37 °C and 5% CO_2_ in darkness. The NRU test was used to check the cytotoxicity of sapropel extracts at three FA concentrations—17.5 μg/mL, 70 μg/mL, and 140 μg/mL; sapropel extract solvent at the proper concentration was used as a control. Sodium laureth sulphate (SLS) (ACS, Sigma-Aldrich, Steinheim, Germany) at 100 μg/mL concentration was used as a positive cytotoxicity control (no uptake of NR dye). Each plate was prepared for chosen extracts and controls at one of the selected concentrations. Then, 25 μg/mL of NR dye was prepared in S5 medium (95% DMEM, 5% FBS). After incubation with test substances, the medium was gently removed and rinsed with 250 μL 1 x PBS, and 250 μL of NR dye was added to each well for 3 h and incubated in an incubator with 90% humidity at 37 °C and 5% CO_2_ in darkness. After 3 h, the NR dye mixture was gently removed via inversion of the plate, all wells were rinsed with 250 μL 1 x PBS, and 100 μL of desorption solution was added and incubated for 20–45 min in darkness in a shaker. The desorption solution consisted of a mixture of 1% glacial acetic acid, 50% ethanol, and 49% distilled water (obtained from EMD Millipore SPR00SIA1US SmartPak Direct-Q 3 (Millipore Sigma, St. Louis, MI, USA). After 20–45 min, the plate was removed from the shaker and left in darkness for 5 min. It was measured spectrophotometrically at 540 nm.

### 2.6. Determination of Changes in Cell Growth Using Cell-IQ^®^

In vitro assessment of the effect of sapropel extracts on cell growth was performed using a Cell-IQ^®^ method (CM Technologies Oy, Tampere, Finland). Cell-IQ^®^ is an integrated real-time monitoring platform for live cell imaging and analysis. Cell-IQ^®^ was used for single-cell populations to determine basic cell population parameters: cell number, cell proliferation, cell death, shape, size, and rates of growth over time using sapropel extract as growth enhancer. Sapropel extracts were tested on BALB/c 3T3 mouse dermal fibroblasts and HaCaT (human adult low-calcium high-temperature keratinocytes) aneuploid immortal human skin cell line at concentrations of 3.5, 7.0, 17.5, 35.0, 70.0, and 140.0 μg/mL, using FA as the control concentration of 700 μg/mL. HaCaT cells represent a spontaneously transformed human epithelial cell line, developed from a long-term primary culture of human adult skin keratinocytes. As it maintains an epidermal differentiation capacity, it was used to determine sapropel extract effectiveness on human skin. The cells were defrosted from −80 °C, cultured at 37 °C in darkness, and kept in a 5% CO_2_ atmosphere for the S10 culture medium. After the defrosting of cells, at least one passage (one reculturing of cells) was performed. BALB/c 3T3 and HaCaT are adherent cells, so they were separated from the surface of the culture dishes using 0.25% trypsin–EDTA solution.

### 2.7. Statistical Analysis

Cell growth was monitored using the Cell-IQ real-time cell monitoring system, and relative cell growth was analysed using GraphPad Prism version 5.0. Two-way ANOVA and Bonferroni post-test statistical significance were determined as * if *p* < 0.05, ** if *p* < 0.01, and *** if *p* < 0.001; the measurement count in each test was six for each sample type.

Antioxidant properties and other characteristics were analysed using Microsoft Excel Professional Plus 2010, and IBM SPSS Statistics version 20 was used for calculations. Differences were considered statistically significant at *p* < 0.05.

## 3. Results

### 3.1. General Characteristics

**Figure 2:**
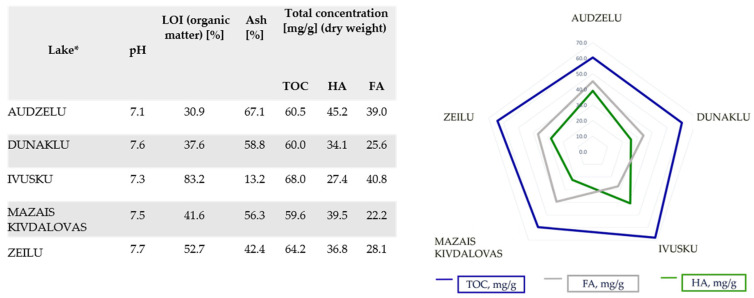
The typical characteristics of sapropel by lake (LOI, loss on ignition; TOC, total organic carbon; HA, humic acid; FA, fulvic acid). * Median values (n = 21 for a lake with three replicates).

The typical characteristics of the analysed sapropel samples are summarised in Figure 2. The moisture content varied from 80 to 93%. The sapropel samples’ pH level was around 7–8, indicating a higher mineral content.

The ash determination and loss-on-ignition (LOI) results are between 30.9 and 83.2%, showing that Ivusku Lake has organic sapropel. Dunaklu and Mazais Kivdalovas Lakes have mixed-type sapropel, Audzelu Lake has silica sapropel, and Zeilu Lake has carbonate sapropel.

### 3.2. TOC, FA, and HA Concentrations

The concentration of FA and HA in sapropel extracts is higher in sapropel with higher TOC. The high organic acid concentration in the lakes can be related to the way in which sapropel forms in lake basins. It is also determined by the formation of organic acids, the degradation of organic matter, and mineralisation processes in the lakes. Figure 2 shows that the highest HA concentrations are in Audzelu Lake (45.2 mg/g), Mazais Kivdalovas Lake (39.5 mg/g), and Zeilu Lake (36.8 mg/g), but the highest FA concentration is in Ivusku Lake (40.8 mg/g). The data show that the ratio between HA and FA is different for separate lakes. As shown in Figure 2, the highest TOC concentration is in Ivusku Lake (68.0 mg/g); the lowest TOC concentration is in Mazais Kivdalovas Lake (59.6 mg/g).

### 3.3. AO Activity

The concentration of organic acids in sapropel extracts was a key factor in determining whether they possess AO or pro-oxidant activity. All samples showed concentration-dependent AO activity; AO parameters were measured for sapropel extract standardised to an FA concentration of 700 μg/mL.

**Figure 3:**
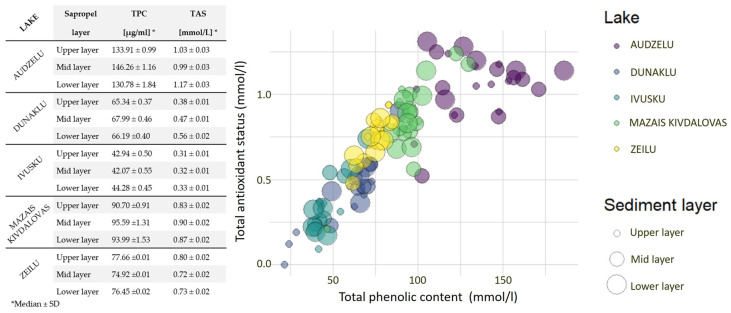
Correlation between total phenolic content and total antioxidant status in different lakes and in different layers.

TAS levels were calculated for each sapropel layer of the lake (n = 21 for each lake with three replicates). No significant differences in the TAS of each sapropel extract from different layers were observed. However, TAS values differ in extracts from separate lakes—the highest values were presented in Audzelu Lake at 1.08 ± 0.03 mmol/L with silica-type sapropel, but the lowest TAS values were found in Ivusku Lake at 0.31 ± 0.01 mmol/L, which has organic-type sapropel. TAS values were found to be higher in sapropel extract samples with higher HA concentrations from Audzelu, Mazais Kivdalovas, and Zeilu Lakes. There is a significant correlation (R^2^ = 0.90) between TAS and HA concentrations in sapropel extracts with the same FA concentration. The investigated extracts show a relatively high polyphenol content range: 42.07–146.26 μg GAL/mL of the sample. The highest TPC was found in Audzelu Lake (146.26 ± 1.16 µg GAL/mL), which has high HA content and TAS, but the lowest was in Ivusku Lake (42.07 ± 0.55 µg GAL/mL). Moreover, there is a substantial correlation between TPC and TAS (R^2^ = 0.93), as shown in Figure 3. Therefore, there is strong correlation between AO activity assessed as both antioxidant status and polyphenol content and HA concentration, and no correlation between AO level and FA concentration.

### 3.4. Neutral Red Uptake

**Figure 4:**
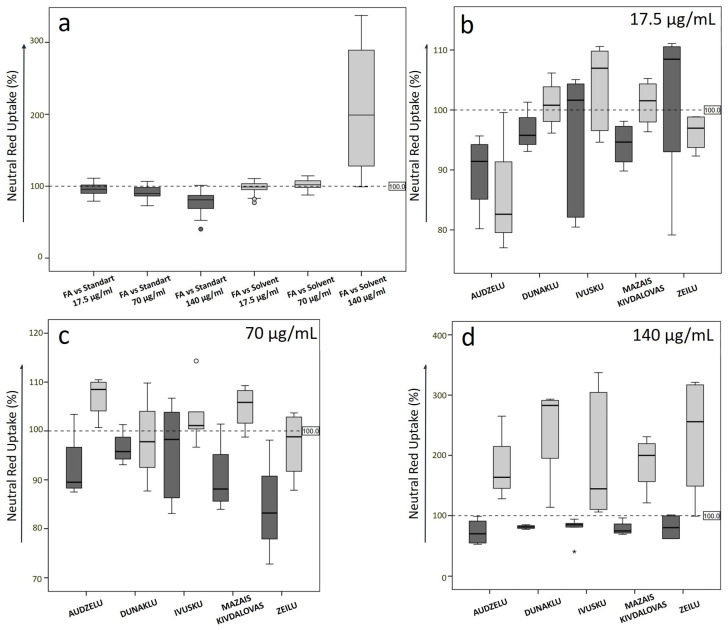
Neutral red uptake by BALB/c 3T3 mouse fibroblasts after exposure to sapropel extract from every lake at 17.5, 70.0, and 140.0 μg/mL concentrations standardised by FA compared to 100% for cells grown in standard medium (S10 control, dark bars) and to solvent control (light bars): (**a**) all lakes together; (**b**) sapropel extract of 17.5 μg/mL concentration by lake; (**c**) 70 μg/mL by lake; and (**d**) 140 μg/mL by lake. Circles indicate outliers, asterisks indicate extreme outliers.

The concentration-dependent cytotoxicity of sapropel extracts was checked via the NRU method. Knowing that the uptake of neutral red dye by live cells can be influenced not only by the tested sapropel extract but also by other substances present in the medium for cell growth, it is important to consider these factors in the analysis of the data. For this reason, the assessment of the results was conducted in two ways: obtained NRU data were recalculated relative to standard medium (S10) control and solvent control to exclude the effect of the solvent in our experiment. Then, both results were analysed and compared to each other (Figure 4).

The NRU data in both recalculation scenarios correlated well only at low concentrations of sapropel extract standardised using FA (FA 17.5 μg/mL, Spearman’s correlation coefficient *r_s_* = 0.492, *p* = 0.023). At higher concentrations, NRU data, compared to S10 and solvent control, did not correlate well, indicating some toxic effects from the solvent (for sapropel extract with FA concentration 70 μg/mL *r_s_* = −0.010, *p* = 0.964; for 140 μg/mL *r_s_* = −0.017, *p* = 0.942).

Sapropel extracts from different lakes have varying effects on NRU at various FA concentrations. Sapropel extracts from all the lakes at the FA concentration of 140 μg/mL showed a noticeable NRU decrease compared to 100% for cells grown in standard medium (S10 control) (Figure 4a). On the other hand, the comparison to solvent control has shown opposite results, with a significant increase in NRU (Figure 4d). This might indicate the effect of solvent toxicity at high concentrations of sapropel extract.

From further analysis for separate lakes, the data in Figure 4b–d demonstrate that NRU after exposure to sapropel extract from Audzelu Lake at 17.5, 70.0, and 140.0 μg/mL FA concentrations was significantly decreased compared to the S10 control. At the same time, it is important to note that compared to the solvent control, NRU for the sapropel extract from Audzelu Lake was increased at 70.0 and 140.0 μg/mL concentration, probably indicating the toxicity of the solvent more than the toxicity of the sapropel extract itself.

For Ivusku and Zeilu Lake sapropel extracts at a low FA concentration (17.5 μg/mL), no harmful properties were noticed compared to the S10 control and they could potentially have beneficial effects that increase NR accumulation by cells, meaning that the cells in these samples are more viable than in the control. Compared to the solvent control, the sapropel extract from Ivusku Lake has shown even better NRU results, but the sapropel extract from Zeilu Lake has worse NRU (Figure 4b).

Sapropel extracts from Dunaklu and Mazais Kivdalova Lakes at low concentrations have shown moderate results compared to S10 and solvent controls but still indicate potential solvent toxicity.

Sapropel extracts at a slightly higher FA concentration of 70 μg/mL showed lower NRU in both scenarios compared to S10 and solvent control, regardless of the lake location. Slightly better NRU was seen only for sapropel extracts from Audzelu, Ivusku, and Mazais Kivdalova Lakes compared with the data for the solvent control.

NRU did not differ significantly when analysed by sapropel extraction depth for all FA concentrations tested. The NRU results slightly decreased between the upper level and the deepest layer at the lowest concentration, as well as at the highest 140 μg/mL concentration. Conversely, each lake slightly increased at a 70 μg/mL concentration.

### 3.5. BALB/c 3T3 and HaCaT Cell Growth

Cell growth changes during exposure to various concentrations of sapropel extracts were observed using the Cell-IQ real-time monitoring system. The cell growth findings demonstrate that at concentrations of 3.5 μg/mL and 7 μg/mL, sapropel extracts do not affect BALB/c 3T3 mouse fibroblasts or HaCaT cell growth during the incubation period; however, extracts from Mazais Kivdalovas Lake and Dunaklu Lake have significant inhibitory effects on HaCaT cell growth if incubated at these low concentrations for longer than 24 h.

**Figure 5:**
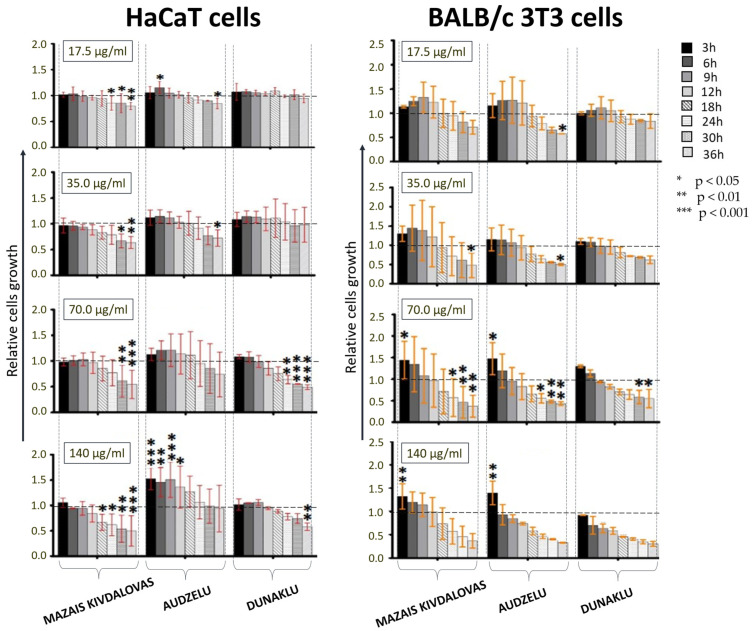
Relative cell growth during 36 h incubation period with sapropel extracts from Mazais Kivdalovas, Audzelu, and Dunaklu Lakes by concentrations of extracts (17.5 µg/mL, 35.0 µg/mL, 70 µg/mL, and 140.0 µg/mL) and cell culture type (HaCaT and BALB/c 3T3 cells).

Figure 5 shows the results of testing with higher concentrations (17.5, 35.0, 70.0, 140 μg/mL) of sapropel extracts. As can be seen, at 17.5 μg/mL concentration, a significant promotion of BALB/c 3T3 cell growth was observed for 12 h, with a significant decrease in growth after 24 h. A decrease in HaCaT cell growth was also observed after incubation with Mazais Kivdalovas Lake sapropel extract for more than 18 h.

At 35 μg/mL concentration, extracts do not change HaCaT cell growth, though Mazais Kivdalovas Lake and Audzelu Lake samples do inhibit cell growth if co-incubation is longer than 24 h (Figure 5). For BALB/c 3T3 cells, a slight increase in cell growth was observed after the exposure of cells to sapropel extract at 35 μg/mL from Mazais Kivdalovas Lake, but only for the first 12 h, then the later notable inhibitory effect appeared. For Audzelu and Dunaklu Lakes, an inhibitory effect of the extract on BALB/c 3T3 cells was also found, but the initial stimulation was less prominent.

At 70 μg/mL concentration, extracts have a significant inhibitory effect on BALB/C 3T3 cell growth if incubated for more than 9 h, i.e., it appeared sooner, especially for extracts from Audzelu and Dunaklu Lakes. For the Mazais Kivdalovas Lake sapropel extract, an initial notable stimulating effect on BALB/c 3T3 was seen, but only for 6 h; later, a significant and more profound inhibitory effect started. HaCaT cell growth was not significantly affected by 12 h of co-incubation with a 70 μg/mL extract of sapropel from Audzelu Lake and for 9 h with an extract from Dunaklu Lake. After this time, a significant inhibitory effect was observed for extracts from both lakes. Sapropel extract from Audzelu Lake at 70 μg/mL concentration had a slight stimulating effect on HaCaT cell growth over the longer period of 18 h.

Co-incubation with a 140 μg/mL extract from Audzelu Lake significantly promoted cell growth for up to 18 h in HaCaT cells and for up to 3 h in 3T3 cells. An extract from Mazais Kivdalovas Lake had a minimal stimulating effect on HaCaT cells but a significant inhibitory effect after 12 h. In BALB/c 3T3 cells, an initial stimulating effect was seen for extracts from Mazais Kivdalovas and Audzelu Lakes, but only for up to 3 h of co-incubation, with a sharp decrease in cell growth afterwards.

## 4. Discussion

In the present study, the sapropel was classified according to the typical characteristics of natural sediments based on the properties (Figure 2) [2]. There are no regulatory acts on quality standards, and there is no international classification for sapropel [3]. However, the international standard “ISO 21426:2018 Annex D: Guidelines for Control Analysis of Peloids and Monitoring” can be used to better understand sapropel sediment evaluation and safety control for use in medical treatments [23]. The investigated sapropel extracts have relatively high AO activity and TPC in various lake samples, which is in accordance with studies reported by Obuka and co-workers [27]. Sapropel extracts show a high polyphenol content, and this has a strong correlation with the TAS, so polyphenols in sapropel extracts are responsible for AO activity [9,28]. The high TPC content may allow sapropel extracts to represent a prospective preparation for the treatment of skin diseases and complex wounds [29,30], as well as for local applications to people suffering from long-healing wounds/injuries (e.g., type II diabetes patients) [8,9], as these wounds do not heal quickly, form scar tissues, and are prone to inflammation.

In order to complete the picture of potential usage in skin applications, the results were analysed from a cosmetic ingredient perspective. The SCCS guidelines indicate that neutral red uptake phototoxicity is a validated in vitro method and its use is mandatory for testing for phototoxic potential [22]. As sapropel extracts contain fulvic and humic acids and they are known as photosensitisers, this test was used to determine toxicity [6,8]. Also, the SCCS guidelines talk about research focusing on using non-animal, human-relevant models, and, subsequently, more human-relevant testing of HaCaT cell growth was carried out. This testing method allowed us a better understanding of sapropel extracts’ effects on human skin and potential wound-healing properties.

The NRU experiment was carried out without the optimisation and adaptation of sapropel extracts for use in mammalian cell cultures, as this was the first phase of assessing whether the selected extracts were harmful (cytotoxic) to cell cultures; the whole purpose was to test sapropel extracts as used in other project activities and to evaluate their potential to reduce cell viability [31,32]. Under the given conditions, 17.5 μg/mL, 70.0 μg/mL, and the highest possible concentration of 140 μg/mL were chosen. However, to acquire this concentration, the medium used for cell culturing was diluted by up to 40% (in the 70.0 μg/mL case, it was 20%), while such a situation provided an opportunity to evaluate NRU under double-stress conditions: diluted media (40% less of the supplements necessary for successful cell growth), a high FA concentration, and a low medium pH. Future experiments should abstain from such dilutions [33,34]. To avoid toxicity in the solvent, finding ways to better control the pH and extract concentration for the cell growth milieu is essential in further studies. It also should be taken into account that while BALB/c 3T3 cells are robust, due to higher extract concentrations, low pH and, perhaps, some other extract/solvent properties, they can lose adherence while performing NRU tests, and, thus, low NRU can be observed in some wells. This was especially relevant (and observed) compared with the solvent control and could be explained by a loss of cell mass due to starvation stress and cell detachment due to high medium dilution and a very low pH [32,35]. It could be hypothesised that under high-starvation/low-pH stress, the high concentration of extract (70 and 140 μg/mL) could help cells maintain viability and adherence to the surface. When interpreting the results, it is important to remember that samples did not have the ideal pH value and consistency for testing in mammalian cell cultures, as these cells are also sensitive to pH change [32]. It could be speculated that perhaps the observed effects of sapropel’s ability to influence NRU were not only due to the biological activity of the extracts but also due to their pH buffering capacity; thus, maybe cell survival was more connected to normalising the pH value in the medium than the biologically active substances in the sapropel extract being able to otherwise improve cell viability [33,36,37]. High concentrations of sapropel extracts made the cytotoxic effect of the solvent visible; when it was excluded from analysis, NRU appeared to be even higher than that of the S10 control.

Regarding higher concentrations, it could be that extracts from some lakes might help cells stay viable under stress conditions for a certain time when cell media are diluted, the pH is not optimal, and there is a high FA concentration. However, evaluating the solvent control to see what type of effect the sapropel extract had without the solvent effect for all samples showed that at 140 μg/mL compared to the solvent, it was remarkably higher, so the solvent plays the main role in toxicity. It could be speculated that sapropel extract containing FA and HA has some properties that reduce starvation stress and help cells maintain viability, similar to the S10 control. However, this hypothesis should be fully tested in different sets of experiments [38].

Moreover, the results that we present here on cell growth indicate that biologically active substances (fulvic acid and humic acid) from sapropel in the short-term (up to 3 h) co-incubation of cells with high concentrations of sapropel extract promote cell growth. Consequently, cell growth is not significantly reduced if incubation with an extract at a high concentration is no longer than 12 h. The results suggest that sapropel extracts have beneficial qualities; for short-term applications, they all could potentially be used in cosmetic applications. However, the further determination of other effects on cells is needed [6,28,29]. It is known that HA has the ability to generate active oxygen from the presence of oxygen, water, and radiation. This process can accelerate wound healing. However, oxygen-driven radicals can cause cell destruction or lipid peroxidation. However, based on HA antioxidant activity, it also has compensation ability; HA solutions produce oxygen only in the amount that is needed, and, at the same time, can restrict peroxidation. This explains why sapropel extracts are effective in lower concentrations, as lower concentrations are optimal for maintaining balance in the system.

Overall, NRU and cell growth data have shown that the biological activity of sapropel extracts can vary a lot depending on the lake location, composition, concentration, and time of exposure. The SCCS Notes of Guidance for the Testing of Cosmetic Ingredients and Their Safety Evaluation recommend general principles for calculating the threshold of toxicology concerns; these calculations also could help determine sapropel extract safety without expensive testing. The calculations represent a pragmatic tool that is based on established principles of systematic adverse event estimation in human health. However, it is not a standalone alternative in cases when the toxicity data availability is limited, so, for future studies, a detailed analysis of natural sapropel samples should be performed before testing on cell cultures to better understand the effects, and non-animal, human-relevant toxicity models are advised. Knowing that sapropel is a natural product with varied contents, sapropel extracts from different locations could have contrasting effects on human cells. For potential use in skin treatment, sapropel extracts with optimal composition and the most beneficial biological effects in cell cultures should be chosen.

Nevertheless, further research must be conducted to find the most promising sapropel extracts for skin applications. Nowadays, there are known compositions with dry sapropel extract in a water-insoluble form for skincare in cases of eczema, dermatitis, and other skin diseases. Sapropel powder extract is added to ointments and other skin care products, but it has limitations when it comes to penetrating the epiderma. Water-soluble skincare products could provide better penetration into the skin and could work on deeper tissues. The current study classifies sapropel extracts based on natural sediment characteristics and explores their potential applications in medical treatments, particularly in skin disease management. Future prospects could involve leveraging international standards to enhance sapropel sediment evaluation and safety control, thereby facilitating their use in medical treatments. Further research could focus on understanding the effects of sapropel extracts on skin cells using non-animal, human-relevant models, and a detailed analysis of natural sapropel samples is recommended before testing on cell cultures to determine the optimal compositions for skin applications. Additionally, exploration into water-soluble sapropel extract formulations may offer improved penetration into the skin for enhanced efficacy in treating skin diseases.

## 5. Conclusions

The FA and HA contents in the sapropel extracts are comparable in the extracts from separate layers and differ depending on the lake because of its origin; the ratio of the FA and HA acids also varies in the separate lakes. The high concentration of FA and HA in the samples indicates the potentially high biological activity of sapropel extract.

The ratio of HA and FA, along with other characteristics of the sapropel, such as pH, organic matter content, ash content, and polyphenolic content, can be used to characterise the sapropel from a particular lake and used as a tool for the identification of the source of sapropel for pharmaceutical and cosmetic product manufacturing.

A strong correlation was found between AO activity (TAS) and HA concentration, as well as TPC.

According to the results for NRU, the sapropel extract with HA and FA does not cause significant harm in cell cultures of human keratinocytes and mouse dermal fibroblasts and could potentially be tested for the development of products intended for humans. Potentially, sapropel might protect the skin from environmental stress due to its AO properties and exhibit beneficial properties during short-term use. Sapropel extracts from the freshwater lakes of the Latgale Upland are expected to be used in the development of cosmetic and pharmaceutical products for application to the skin. Furthermore, this could increase the use of locally available natural resources as one of the world’s priorities.

## 6. Patents

We have a patent of the Republic of Latvia LR 15514 A. A61Ql9/00, A61K8/02. A. Auce, A. Klavina, I. Vanadzins, I. Pavlovska, A.Silova, L. Dobkevica, L.Komarovska, B. Silamikele: sapropel extract water soluble gel and the method for its preparation. Pat. Appl. P-19-53, 29.11.2019. Publ. 20.07.2020.

## Data Availability

The datasets used and analysed during the current study are available from the corresponding author on reasonable request.

## Abbreviations

The following abbreviations are used in this manuscript:

ABTS	2,2′-azino-bis(3-ethylbenzothiazoline-6-sulfonic acid)
AO	antioxidant activity
AR	analytical-reagent (AR)-grade chemicals are chemicals that meet the specifications outlined for analytical applications in laboratories. These chemicals are of high purity, typically exceeding 95 percent, with impurities specified and controlled to ensure accuracy and reliability in analytical procedures.
BALB/c 3T3	Bagg Albino mouse fibroblast cells
DDE	dichlorodiphenyldichloroethylene
DDT	dichlorodiphenyltrichloroethane
DMEM	Dulbecco’s Modification of Eagle’s Medium
FA	fulvic acid
FBS	foetal bovine serum
HA	humic acid
HaCaT	human adult low-calcium high-temperature keratinocytes (HaCaT cells)
LOI	loss on ignition
NRU	neutral red uptake
OECD	Organisation for Economic Co-operation and Development
PBS	phosphate-buffered saline
SCCS	Scientific Committee on Consumer Safety
TAS	total antioxidant status
TOC	total organic carbon
TPC	total polyphenolic content

## References

[1] Sharypov V.I., Beregovtsova N.G., Baryshnikov S.V., Rudkovsky A.V. (2015). The Study of Ethanol Extracts Composition of Organic (Kachkulnya Lake) and Organomineral (Barchin Lake) Sapropels from Novosibirsk Region. J. Sib. Fed. Univ. Chem..

[2] Stankevica K., Klavins M. (2014). Sapropel and Its Application Possibilities. Mater. Sci. Appl. Chem..

[3] Pavlovska I., Klavina A., Auce A., Vanadzins I., Silova A., Komarovska L., Silamikele B., Dobkevica L., Paegle L. (2020). Assessment of Sapropel Use for Pharmaceutical Products According to Legislation, Pollution Parameters, and Concentration of Biologically Active Substances. Sci. Rep..

[4] Vanadziņš I., Mārtiņsone I., Kļaviņa A., Komarovska L., Auce A., Dobkeviča L., Sprūdža D. (2022). Sapropel—Mining Characteristics and Potential Use in Medicine. Proc. Latv. Acad. Sci. Sect. B Nat. Exact Appl. Sci..

[5] Sanchez-Espejo R., Aguzzi C., Cerezo P., Salcedo I., Lopez-Galindo A., Viseras C. (2014). Folk Pharmaceutical Formulations in Western Mediterranean: Identification and Safety of Clays Used in Pelotherapy. J. Ethnopharmacol..

[6] van Rensburg C.E.J. (2015). The Antiinflammatory Properties of Humic Substances: A Mini Review. Phytother. Res..

[7] de Melo B.A.G., Motta F.L., Santana M.H.A. (2016). Humic Acids: Structural Properties and Multiple Functionalities for Novel Technological Developments. Mater. Sci. Eng. C.

[8] Jacob K.K., Prashob Peter K.J., Chandramohanakumar N. (2019). Humic Substances as a Potent Biomaterials for Therapeutic and Drug Delivery System-a Review. Int. J. Appl. Pharm..

[9] Winkler J., Ghosh S. (2018). Therapeutic Potential of Fulvic Acid in Chronic Inflammatory Diseases and Diabetes. J. Diabetes Res..

[10] Gomes C., Carretero M.I., Pozo M., Maraver F., Cantista P., Armijo F., Legido J.L., Teixeira F., Rautureau M., Delgado R. (2013). Peloids and Pelotherapy: Historical Evolution, Classification and Glossary. Appl. Clay Sci..

[11] Jarukas L., Ivanauskas L., Kasparaviciene G., Baranauskaite J., Marksa M., Bernatoniene J. (2021). Determination of Organic Compounds, Fulvic Acid, Humic Acid, and Humin in Peat and Sapropel Alkaline Extracts. Molecules.

[12] Dolmaa G., Tserenpil S., Ugtakhbayar O., Shevchenko S., Kliba L., Voronkov M. (2011). Characterization and Organic Compounds in Peloids from Mongolia. Proc. Mong. Acad. Sci..

[13] Tserenpil S., Dolmaa G., Voronkov M.G. (2010). Organic Matters in Healing Muds from Mongolia. Appl. Clay Sci..

[14] Alexandrova G.P., Dolmaab G., Tserenpil S., Grishenko L.A., Sukhov B.G., Regdel D., Trofimov B.A. (2013). A New Humic Acid Preparation with Addition of Silver Nanoparticles. Functions of Natural Organic Matter in Changing Environment.

[15] Spaccini R., Cozzolino V., Di Meo V., Savy D., Drosos M., Piccolo A. (2019). Bioactivity of Humic Substances and Water Extracts from Compost Made by Ligno-Cellulose Wastes from Biorefinery. Sci. Total Environ..

[16] Bos J.D., Meinardi M.M.H.M. (2000). The 500 Dalton Rule for the Skin Penetration of Chemical Compounds and Drugs. Exp. Dermatol..

[17] McKirdy D.M., Spiro B., Kim A.W., Brenchley A.J., Hepplewhite C.J., Mazzoleni A.G. (2013). Environmental Significance of Mid- to Late Holocene Sapropels in Old Man Lake, Coorong Coastal Plain, South Australia: An Isotopic, Biomarker and Palaeoecological Perspective. Org. Geochem..

[18] Canellas L.P., Olivares F.L., Aguiar N.O., Jones D.L., Nebbioso A., Mazzei P., Piccolo A. (2015). Humic and Fulvic Acids as Biostimulants in Horticulture. Sci. Hortic..

[19] Wang B., Wu L., Chen J., Dong L., Chen C., Wen Z., Hu J., Fleming I., Wang D.W. (2021). Metabolism Pathways of Arachidonic Acids: Mechanisms and Potential Therapeutic Targets. Signal Transduct. Target. Ther..

[20] Mirza M.A., Agarwal S.P., Rahman M.A., Rauf A., Ahmad N., Alam A., Iqbal Z. (2011). Role of Humic Acid on Oral Drug Delivery of an Antiepileptic Drug. Drug Dev. Ind. Pharm..

[21] Klavina A., Auce A., Pavlovska I., Vanadzins I. (2020). Freshwater Sapropel: Biologically Active Components and Methods of Extraction. Proc. CBU Nat. Sci. ICT.

[22] Scientific Committee on Consumer Safety (2023). SCCS Notes of Guidance for the Testing of Cosmetic Ingredients and Their Safety Evaluation.

[23] (2018). Annex D: Guidelines for Control Analysis of Peloids and Monitoring.

[24] (2005). Determination of Substances Characteristic of Green and Black Tea—Part1: Content of Total Polyphenols in Tea—Colorimetric Method Using Folin-Ciocalteu Reagent.

[25] Blainski A., Lopes C.G., Palazzo de Mello J.C. (2013). Application and Analysis of the Folin Ciocalteu Method for the Determination of the Total Phenolic Content from *Limonium Brasiliense* L. Molecules.

[26] OECD (2019). Test No. 432: In Vitro 3T3 NRU Phototoxicity Test. OECD Guidelines for the Testing of Chemicals, Section 4.

[27] Obuka V., Boroduskis M., Ramata-Stunda A., Klavins L., Klavins M. (2018). Sapropel Processing Approaches towards High Added-Value Products. Agron. Res..

[28] Wang C., Wang Z., Peng A., Hou J., Xin W. (1996). Interaction between Fulvic Acids of Different Origins and Active Oxygen Radicals. Sci. China C Life Sci..

[29] Hoang H.T., Moon J.Y., Lee Y.C. (2021). Natural Antioxidants from Plant Extracts in Skincare Cosmetics: Recent Applications, Challenges and Perspectives. Cosmetics.

[30] Guimarães I., Baptista-Silva S., Pintado M., Oliveira A.L. (2021). Polyphenols: A Promising Avenue in Therapeutic Solutions for Wound Care. Appl. Sci..

[31] ICCVAM (2006). ICCVAM-Recommended Test Method Protocol BALB/c 3T3 NRU Cytotoxicity Test Method. ICCVAM Test Method Evaluation Report Appendix.

[32] Phelan K., May K.M. (2016). Basic Techniques in Mammalian Cell Tissue Culture. Curr. Protoc. Toxicol..

[33] Repetto G., del Peso A., Zurita J.L. (2008). Neutral Red Uptake Assay for the Estimation of Cell Viability/Cytotoxicity. Nat. Protoc..

[34] The Food and Drug Administration (2015). S10 Photosafety Evaluation of Pharmaceuticals. Fed. Regist..

[35] The Food and Drug Administration (2012). S6 Addendum to Preclinical Safety Evaluation of Biotechnology-Derived Pharmaceuticals. Fed. Regist..

[36] Philippeos C., Hughes R.D., Dhawan A., Mitry R.R. (2012). Introduction to Cell Culture. Methods Mol. Biol..

[37] Phelan K., May K.M. (2017). Mammalian Cell Tissue Culture. Curr. Protoc. Hum. Genet..

[38] Jurcsik I. (1994). Possibilities of Applying Humic Acids in Medicine (Wound Healing and Cancer Therapy). Humic Substances in the Global Environment.

